# Carbonization and oxidation of metal–organic frameworks based on 1,4-naphthalene dicarboxylates

**DOI:** 10.1088/1468-6996/16/5/054203

**Published:** 2015-10-06

**Authors:** Jiun-Jen Chen, Ya-Ting Chen, Duraisamy Senthil Raja, Yu-Hao Kang, Pen-Chang Tseng, Chia-Her Lin

**Affiliations:** 1 Green Energy & Environment Research Laboratories, Industrial Technology Research Institute, Hsinchu 310, Taiwan; 2 Department of Chemistry, Chung Yuan Christian University, Chung-Li 320, Taiwan; 3 R&D Center for Membrane Technology & Research Center for Structure of Matter, Chung Yuan Christian University, Chung-Li 320, Taiwan

**Keywords:** nanoporous carbon, metal oxide nanoparticle, metal–organic frameworks

## Abstract

Three new isostructural metal–organic frameworks (MOFs), [V(OH)(NDC)] (**1**), [Cr(OH)(NDC)] (**2**), and [Ga(OH)(NDC)] (**3**) have been synthesized hydrothermally using 1,4-naphthalene dicarboxylate (NDC) as the linker. These MOFs (**1**, **2** and **3**) have been used as a template for the synthesis of metal-oxide-inserted nanoporous carbon materials. The newly synthesized MOFs and the resulting porous carbon hybrid functional materials have been characterized using powder x-ray diffraction, scanning electron microscopy, transmission electron microscopy, and energy dispersive x-ray spectroscopic analysis. Results show that compounds **2** and **3** form their respective metal oxide nanoparticles on the surface of the carbon materials during carbonization at 800 °C. The gas sorption properties of the new MOFs and their corresponding carbon frameworks have been reported.

## 1. Introduction

Metal–organic frameworks (MOFs) have seen a rapid growth in the past decade because of their interesting structural properties and potential applications in different fields [1–7]. Among the strategies applied for the synthesis of MOFs, the isoreticular concepts provide many advantages for the structural design and functional applications of MOFs [8, 9].

On the other hand, since porous carbon materials have a high surface area, tunable porous structure, high thermal stability, chemical stability, and adsorption ability, they are widely used in a variety of applications such as adsorbents [10], separation membranes [11], super capacitors [12], sensors [13], and so on. Porous carbon materials can be prepared in many ways, such as activation, carbonization of polymers, and template synthesis [14–17]. In this regard, inorganic porous materials, such as mesoporous silica and zeolites, have been successfully used as templates for the synthesis of porous carbon functional materials in recent years [15, 16]. Each method has its own merits for the formation of carbon frameworks with controlled pore size and/or enhanced surface area, which are considered to be the major factors for functional applications [18, 19].

Since the metal centers and organic bridging linkers are well organized in MOFs, they were recently shown to be suitable precursors for constructing porous carbon frameworks [10]. Further, due to the confinement effect of MOFs, the regular arranged metal centers can be transformed into metal/metal oxide nanoparticles during the carbonization process, while the organic bridging linkers tend to form fine porous carbon frameworks [20]. So, this *in situ* approach leads to dispersion of the metal/metal oxide nanoparticles throughout the carbon frameworks. It is interesting to note that the resulting carbon frameworks have prevented the metal/metal oxide nanoparticles from aggregating [20].

Although many carbon nanocomposite materials have already been synthesized using MOFs as precursors in recent years [10, 12, 13], the design and construction of carbon frameworks from MOF precursors are still in a developing stage. Also, the direct synthesis of carbon–metal oxide hybrid materials from a MOF as a single precursor is still rare [20, 21]. With this background in mind, we have synthesized three new isostructural MOFs ([M(OH)(NDC)] (**1**–**3**), where M = V, Cr, and Ga respectively) having the same organic linker (1,4-naphthalene dicarboxylate, NDC) but different metal cations (V^3+^, Cr^3+^, Ga^3+^), and utilized them as precursors for the synthesis of a nanoporous carbon matrix through a simple one-step thermal conversion without using any additional carbon source.

## 2. Experimental section

All reagents were commercially available and used as received without further purification. The hydrothermal reactions were carried out by heating the reaction mixtures in 23 mL Teflon-lined digestion bombs to the desired temperature under autogenous pressure, followed by slow cooling at the rate of 6 °C h^−1^ to room temperature. The phase purity of all the compounds was examined by powder x-ray diffraction (PXRD) using a Bruker D2 PHASER instrument. Elemental analyses were carried out using an Elementar vario EL III Heraeus CHNOS Rapid F002 instrument. Thermal gravimetric analyses (TGA) using a DuPont TA Q50 analyser were performed on powder samples under flowing N_2_ with a heating rate of 10 °C min^−1^. The gas sorption isotherms were measured at 77 K for N_2_, and 273 and 298 K for CO_2_ using an ASAP 2020 system of Micromeritics. Ultrahigh purity grade CO_2_, N_2_, and He were used as received. Before the gas sorption measurements, the sample was initially dehydrated at 423 K for 24 h under vacuum. Scanning electron microscopy (SEM, using a JEOL JEM-7600F instrument) and transmission electron microscopy (TEM, using a JEM-2010 instrument) were employed to characterize the morphology.

In a typical synthesis of the MOFs, the mixture of VCl_3_ (0.154 g, 1.0 mmol), H_2_NDC (0.108 g, 0.5 mmol), and H_2_O (10 mL) was placed in a 23 mL Teflon autoclave and then heated at 180 °C for 1 day. A light-green powder of **1** was obtained (yield: 0.115 g). Elemental analysis, found/calculated: C, 47.96/48.02; H, 3.11/3.02% for **1**·H_2_O. MOF **2** was synthesized by same procedure with CrCl_3_·6H_2_O (0.266 g, 1.0 mmol), H_2_NDC (0.108 g, 0.5 mmol), and H_2_O (5 mL) at 220 °C for 3 days. A pale-green powder of **2** was obtained (yield: 0.142 g). Elemental analysis, found/calculated: C, 38.07/38.61; H, 4.00/4.60% for **2**·5H_2_O. A similar reaction mixture ratio and conditions as those used for the synthesis of **1** were used for the synthesis of **3** except that of Ga(NO_3_)_3_·xH_2_O instead of VCl_3_. A white powder of **3** was obtained (yield: 0.126 g). Elemental analysis, found/calculated: C, 40.60/40.60; H, 3.19/3.69% for **3**·3H_2_O.

The synthesis of porous carbon framework materials involves simple one-step direct carbonization using the synthesized MOFs as precursors. The MOFs (0.200 g) were taken in a silica boat and then placed in a tube furnace and heated from room temperature to 800 °C under N_2_ gas with a heating rate of 5 °C min^−1^ to carbonize the MOFs. After reaching the target temperature (800 °C), the temperature was maintained at 800 °C for 5 h, after that cooled down to room temperature with a cooling rate of 1 °C min^−1^. The final black colored powder products were further characterized.

## 3. Results and discussion

**Figure 1.**
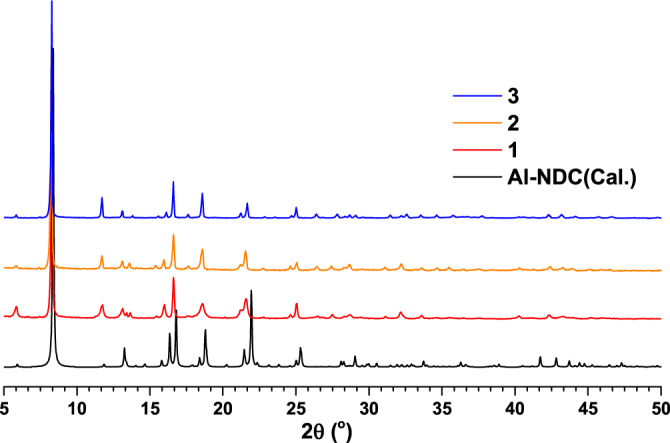
The comparison of PXRD patterns of newly synthesized MOFs, **1**, **2** and **3** with a simulated PXRD pattern of reported Al-NDC.

The simple hydrothermal reactions of H_2_NDC (0.5 mmol) with the corresponding metal salts (1.0 mmol) yielded the new MOFs. The new MOFs have initially been characterized by elemental analysis which agreed well with theoretical values. The phase purity of the bulk materials was independently confirmed by PXRD measurements (figure 1). On comparing the PXRD patterns of **1**–**3** with one another showed that the three new MOFs are isostructural to each other. Further comparison of these PXRD patterns with calculated PXRD patterns of reported aluminum naphthalenedicarboxylate (Al-NDC) MOF material [22] confirms that these new MOFs (**1**–**3**) have the same type of structural architecture as Al-NDC (isostructural).

**Figure 2.**
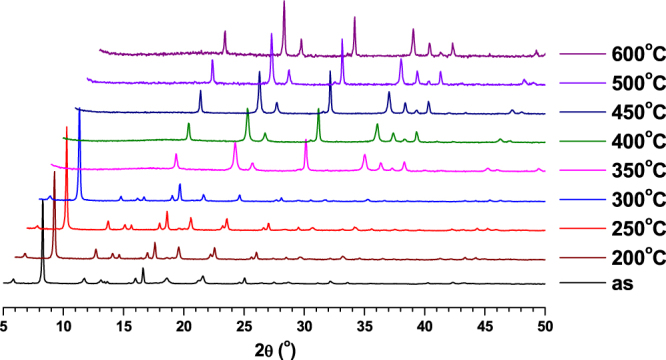
PXRD patterns of **1** at various temperatures.

**Figure 3.**
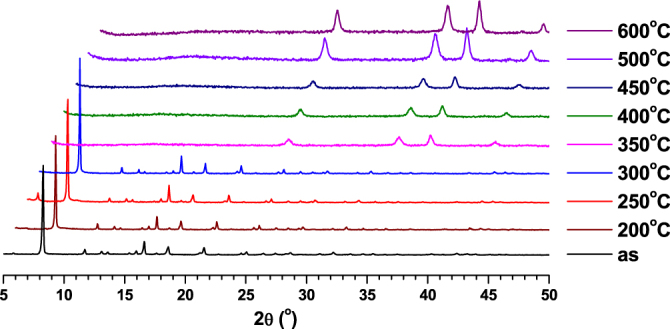
PXRD patterns of **2** at various temperatures.

**Figure 4.**
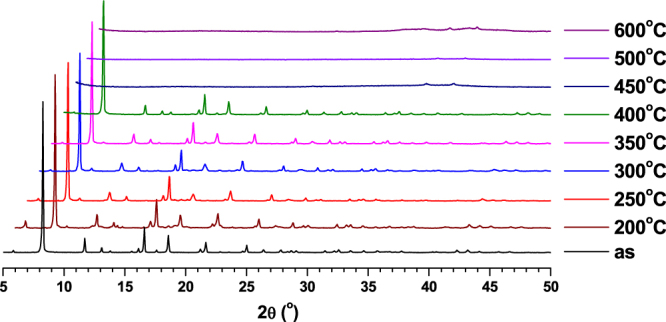
PXRD patterns of **3** at various temperatures.

The TGA measurements for the compounds were performed under N_2_ atmosphere and the results are shown in figure S1. The slight weight loss observed initially (up 80 °C) for compounds **2** and **3** may be due to the presence of small lattice water molecules in it. TGA studies indicated that all the compounds are stable up to 300 °C. This was further confirmed through *in situ* PXRD measurements at various temperatures for all the compounds (figures 2–4). Further, the compounds were heated at various temperatures for one hour, and the corresponding images are given in figures S2–S4. The compounds showed a stable color up to 300 °C owing to their high thermal stability. The calcined products of **1**–**3** at 600 °C were characterized using PXRD measurements and compared with calculated PXRD patterns of their corresponding metal oxides (figures S5–S7). The results indicated that the compounds **1**, **2** and **3** were converted to their corresponding metal oxides (V_2_O_5_, Cr_2_O_3_ and Ga_2_O_3_ respectively) during calcination at 600 °C for one hour.

**Figure 5.**
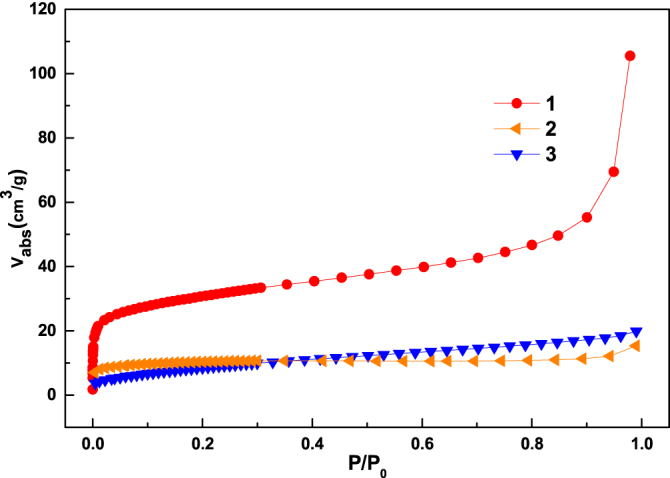
The N_2_ gas adsorption isotherms for **1**–**3** at 77 K.

The porosity of the new MOFs has been analyzed using N_2_ gas sorption measurements at 77 K (figure 5) and the results are shown in table 1. The results indicated that compound **1** has a better pore structure than **2** and **3** (which showed a non-porous nature and adsorption takes place at the external surface). However, the CO_2_ gas sorption measurements showed that compounds **2** and **3** have a porous nature.

**Table 1.** The N_2_ gas sorption measurement results for **1**–**3**. BET stands for Brunauer–Emmett–Teller.

MOFs	BET surface area (m^2^ g^−1^)	Langmuir surface area (m^2^ g^−1^)	Total pore volume (cm^3^ g^−1^) *P*/*P*_o_ ∼ 0.99
**1**	107	292	0.53
**2**	33	48	0.02
**3**	31	45	0.03

**Figure 6.**
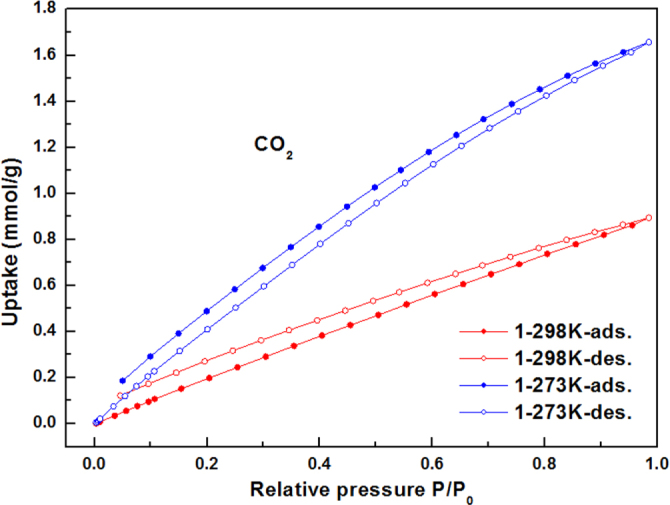
The CO_2_ gas sorption isotherms for **1** at 273 K and 298 K.

**Figure 7.**
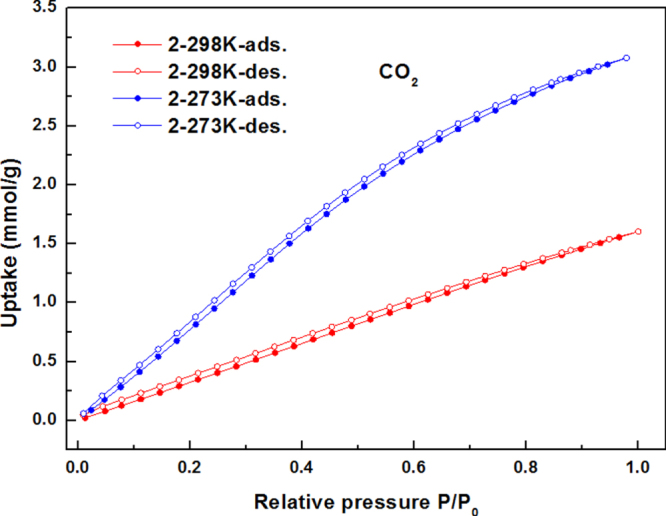
The CO_2_ gas sorption isotherms for **2** at 273 K and 298 K.

**Figure 8.**
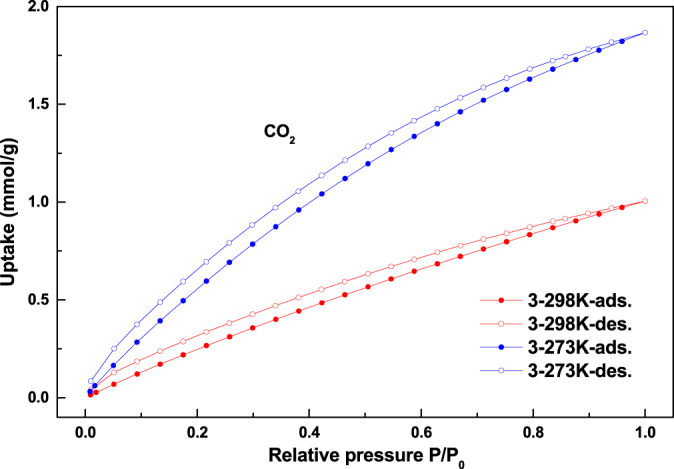
The CO_2_ gas sorption isotherms for **3** at 273 K and 298 K.

The CO_2_ gas sorption isotherms for compounds **1**–**3** at 273 and 298 K are given in figures 6–8. Compounds **1**–**3** showed CO_2_ adsorption of 1.65 mmol g^−1^, 3.07 mmol g^−1^, and 1.86 mmol g^−1^, respectively, at 273 K, 1 atm; whereas 0.89 mmol g^−1^, 1.60 mmol g^−1^, and 1.00 mmol g^−1^ of CO_2_ adsorption have been observed for compounds **1**–**3**, respectively, at 298 K and 1 atm.

**Figure 9.**
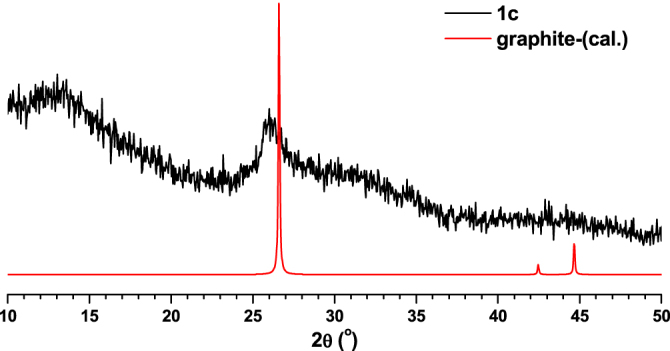
Comparison of PXRD pattern of **1c** with calculated PXRD pattern of graphite.

**Figure 10.**
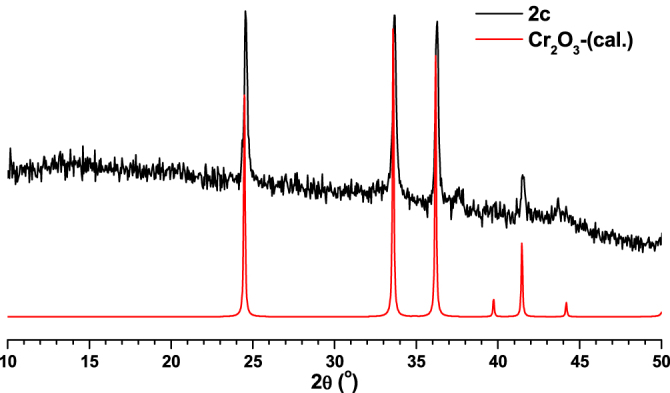
Comparison of PXRD pattern of **2c** with calculated PXRD pattern of Cr_2_O_3_.

**Figure 11.**
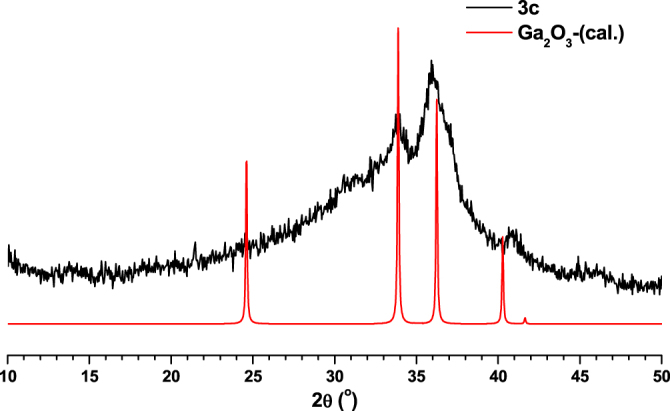
Comparison of PXRD pattern of **3c** with calculated PXRD pattern of Ga_2_O_3_.

In order to study the application possibilities of compounds **1**–**3**, they were used as templates to synthesise nanoporous carbon materials. In a typical carbonization procedure, MOFs (**1**–**3**) were calcined at 800 °C under nitrogen gas flow for 5 h. The final products were characterized with the aid of various techniques. The carbonized products from **1**, **2** and **3** are designated as **1c**, **2c** and **3c** respectively. Compounds **1c**–**3c** were initially characterized using PXRD measurements which indicated that **1c** has a disordered-oriented graphitic type structure (figure 9). At the same time, the PXRD patterns matching **2c** with Cr_2_O_3_ (figure 10) and **3c** with Ga_2_O_3_ (figure 11) suggested the presence of their corresponding metal oxide particles in the synthesized carbon hybrid materials.

**Figure 12.**
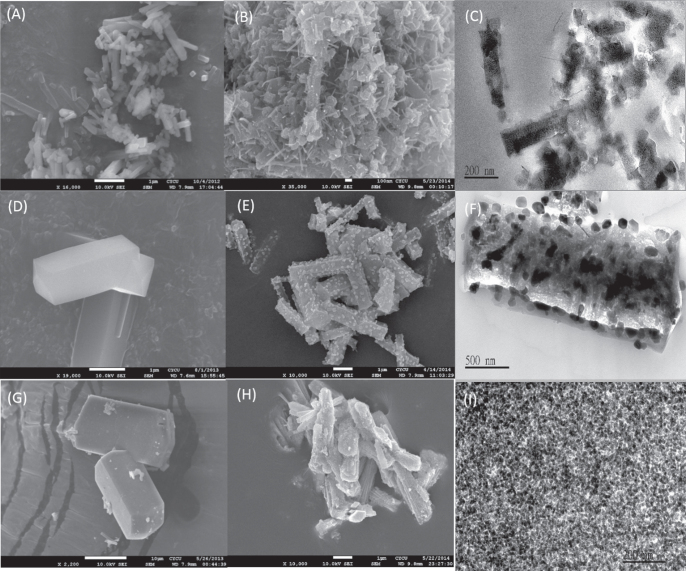
The SEM images of (A) **1**, (B) **1c**, (D) **2**, (E) **2c**, (G) **3**, and (H) **3c**; the TEM images of (C) **1c**, (F) **2c**, and (I) **3c**.

The comparison SEM images of **1**, **2** and **3** with **1c**, **2c** and **3c** respectively (figure 12) suggested that the obtained nanoporous carbons retained a typical crystal morphology similar to that of the parent MOFs. Further, the SEM images of **2c** and **3c** revealed that the surface of nanoporous carbon samples embedded with their corresponding metal oxide nanoparticles. The SEM observations were confirmed by TEM images of the carbon materials (figures 12(C), (F) and (I)). The compositions of the carbon matrix and the metal oxide nanoparticles were confirmed by energy dispersive x-ray spectroscopy (EDS) analysis and the results are given in figures S8–S10.

**Figure 13.**
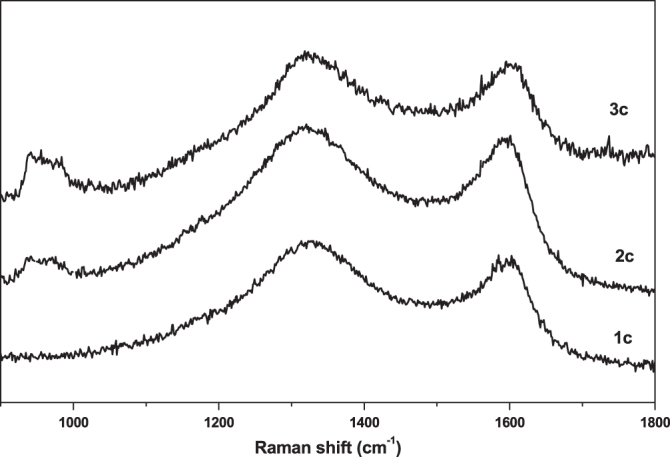
Raman spectra of the obtained nanoporous carbon samples.

Further investigation of the local structure of the synthesized carbon materials was achieved by Raman spectroscopy. Raman spectra of the obtained carbon samples are shown in figure 13, exhibiting D and G bands centered around 1320 cm^−1^ and 1600 cm^−1^, respectively, which may be due the disordered carbon structures and the stretching vibrations in opposite directions of two carbon atoms in a graphene sheet. The relative ratios of the G band to the D band (*I*_G_/*I*_D_) were found to be 1.15, 1.08, and 1.03 for **1c**, **2c** and **3c**, respectively, suggesting that the graphene sheets were not well developed in all samples and the local carbon structures contained both graphitic and disordered carbon atoms [12].

**Figure 14.**
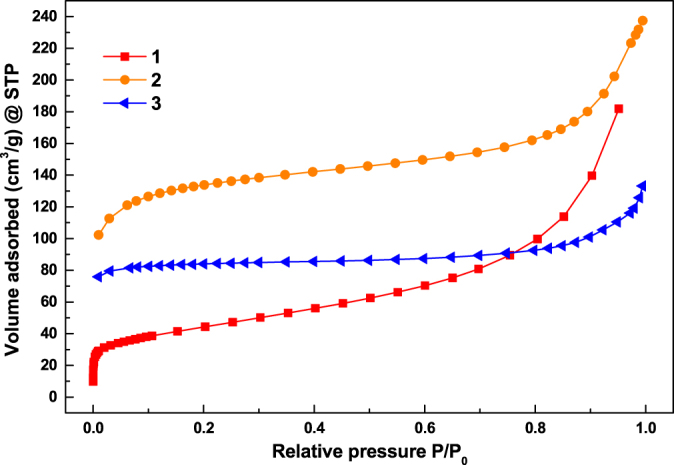
The N_2_ gas adsorption isotherms for **1c**–**3c** at 77 K.

The nitrogen gas adsorption analysis was used to further characterize the nanoporous structure of **1c**, **2c** and **3c** (figure 14). The shape of the isotherms indicated the existence of both micropores and mesopores. The steep increase at low relative pressure indicates the presence of micropores. The isotherms showed a small hysteresis which is typical for the presence of spherical mesopores randomly connected with weak microporosity. The analyzed pore characteristics of the carbon materials are summarized in table 2. Further, the pore size distribution results for **1c**–**3c** obtained using the density functional theory (DFT) method are shown in figure S11. The results indicated that **1c** mostly shows a mesoporous nature, which may be due to the lower stability of the microporous structure of **1** during carbonization, whereas compounds **2c** and **3c** showed some kind of microporosity along with their mesoporous nature. There are a large number of pores of different sizes (2 ∼ 20 nm) observed for all three compounds. Overall, it is interesting to note that the pore structures of the carbon materials are better than those of their parent MOF materials, but are not necessarily better than those reported in the literature. Since the pore structure and surface area of the resultant carbon materials can be tuned simply by changing the calcination temperature, the experimental carbonization temperature (800 °C) may not be the ideal one to make the best porous carbon materials from our new MOFs. Hence, although compound **1** has the highest surface area among these new MOFs, its derived carbon material **1c** has a lower surface area than the other carbon materials (see tables 1 and 2).

**Table 2.** Pore characteristics of the obtained nanoporous carbons.

Materials	BET surface area (m^2^ g^−1^)	Langmuir surface area (m^2^ g^−1^)	Total pore volume (cm^3^ g^−1^) *P*/*P*_o_ ∼ 0.99
**1c**	43	75	0.14
**2c**	435	620	0.36
**3c**	274	373	0.19

## 4. Conclusions

In summary, three new isostructural MOFs based on an NDC linker have been prepared and characterized. Using these MOFs as precursor materials, metal oxide nanoparticle inserted nanoporous carbons were prepared by simple direct carbonization without using any additional carbon sources. The PXRD, SEM, TEM, EDS and nitrogen sorption measurements confirm the dispersion of metal oxide nanoparticles on the resulting nanoporous carbon materials. Further application studies of these metal oxide nanoparticle inserted porous carbon hybrid functional materials are in progress.
